# Prediction of miRNA targets by learning from interaction sequences

**DOI:** 10.1371/journal.pone.0232578

**Published:** 2020-05-05

**Authors:** Xueming Zheng, Long Chen, Xiuming Li, Ying Zhang, Shungao Xu, Xinxiang Huang

**Affiliations:** 1 Department of Biochemistry and Molecular Biology, School of Medicine, Jiangsu University, Zhenjiang, Jiangsu, P. R. China; 2 Department of Clinical Laboratory, the First People’s Hospital of Zhangjiagang, Zhangjiagang, Jiangsu, P. R. China; 3 School of Computer Science, Jiangsu University of Science and Technology, Zhenjiang, Jiangsu, P. R. China; Sun Yat-Sen University, CHINA

## Abstract

MicroRNAs (miRNAs) are involved in a diverse variety of biological processes through regulating the expression of target genes in the post-transcriptional level. So, it is of great importance to discover the targets of miRNAs in biological research. But, due to the short length of miRNAs and limited sequence complementarity to their gene targets in animals, it is challenging to develop algorithms to predict the targets of miRNA accurately. Here we developed a new miRNA target prediction algorithm using a multilayer convolutional neural network. Our model learned automatically the interaction patterns of the experiment-validated miRNA:target-site chimeras from the raw sequence, avoiding hand-craft selection of features by domain experts. The performance on test dataset is inspiring, indicating great generalization ability of our model. Moreover, considering the stability of miRNA:target-site duplexes, our method also showed good performance to predict the target transcripts of miRNAs.

## 1. Introduction

MicroRNAs (miRNAs) are a class of short (approximately 21-nucleotide), non-coding RNAs which can regulate gene expression at the post-transcriptional level. Upon loading into the Argonaute (Ago) proteins [1], which are the catalytic components of the RNA-induced silencing complex (RISC) [2], miRNAs interact with the target mRNAs. These interactions result in mRNA repression, destabilization and thus prevent the target genes from producing functional peptides and proteins. There are more than 2,000 annotated humans miRNAs deposited in miRBase (http://www.mirbase.org/) [3]. Because of the extensive targets, miRNAs are involved in a variety of cellular pathways, from development to pluripotency to oncogenesis and so on [4–7]. The induction of gene silencing and repression via miRNAs typically requires complementary base pairing between specific regions of the miRNA and its target mRNA. Canonical miRNA-mRNA interactions require the target site complementarity to the seed sequence, nucleotides 2–8, of the miRNA [8]. However, there are many examples of functional miRNA-mRNA interactions that occur without perfect seed pairing, indicating the non-canonical interactions [9,10].

There are multiple experimental techniques available to identify targets of miRNAs. Through miRNA-overexpression studies combined by gene-expression analysis, a large number of miRNA:target-gene interactions have been identified [11,12]. Due to the complexity of regulation network, many affected genes may not be the direct targets of miRNAs. Recently, other methods are developed to investigate the direct interaction of miRNA and target sites, such as CLIP-seq (cross-linking immunoprecipitation with sequencing), PAR-CLIP (photoactivatable-ribonucleoside-enhanced CLIP), iCLIP (individual-nucleotide resolution CLIP) etc [13–16]. All these methods can catch the miRNA:target hybrids in the RISC complex and identify lots of miRNA:target-site interactions through high-throughput sequencing.

In addition to experiment techniques, there are lots of computational prediction algorithms developed to predict miRNA targets such as TargetScan, miRTarget3, and miRWalk etc [17–19]. Most methods are mainly based on attributes of the mRNA sequence itself, thermodynamic stability of the miRNA-mRNA duplex, evolutionary conservation, or statistical inference based machine learning [20]. Recently, some deep learning (DL)-based approaches were developed to predict miRNA target sites and/or transcripts [21–23]. But, these methods also depended heavily on the hand-crafted features of the miRNA-target duplex. Besides the deep learning methods to predict the mirna-targets, there are other method to be used for the similar task [21]. Also, it has been shown that the finding such regulatory relationship can be used for cancer subtyping [24]. Because of not fully understood rules that govern miRNAs targeting process and different training datasets for different algorithms, there is limited overlap between the targets that are predicted by various programs. So, it is still a challenge to develop more reliable computation methods based on more accurate miRNA:target datasets.

Deep learning (DL), which can autonomously learn and identify patterns from raw data, has been shown to be an effective method for classification tasks in domains with complex feature representation [25]. Convolutional neural networks (CNNs) are characteristic of convolution layers which can automatically extract features from input datasets and have showed great success in image recognition [26–28]. The convolution layer, consisting of a combination of linear convolution operation and nonlinear activation function, is usually followed by a pooling layer which provides a typical down-sampling operation such as max-pooling [29]. Through using multiple convolution and pooling layers, CNN models can learn the patterns from low to high levels in the training dataset [30].

Lots of CNN architectures have been developed to address biological problems and showed to be successful [31,32]. Here, we designed a multilayer convolutional neural network to predict the target sites of miRNAs without need of feature extraction in advance. To the best of our knowledge, we first applied CNN to extract complex features from raw sequences of miRNA:target-site duplex, which were used for prediction of miRNA targets. Our method can also be used to predict the target gene of miRNAs through scanning the full length of gene transcripts.

## 2. Materials and methods

### 2.1 Datasets preparation

#### MiRNA:Target-site datasets

To get a more reliable dataset, only the experiment-validated direct interactions of miRNAs and targets were collected. The positive miRNA:target data were downloaded from three sources: 1. Study of miRNA interactome by CLASH (crosslinking, ligation, and sequencing of hybrids) in HEK293 cells [33]. 2. Study of miRNAs to their target sites in *C*. elegans using modified CLIP methodology and re-analysis of published mammalian AGO-CLIP data for miRNA-chimeras yielded ~17,000 miRNA:target-site interactions [34]. 3. miRNA:target-site interaction data in MirTarBase with strong experimental evidence (immunoblot, luciferase reporter assay, qRT-PCR) [35]. The miRNA sequences were retrieved from miRBase [36]. All the data were merged followed by removing the duplicates of miRNA:target-site sequences and the concatenated miRNA:target chimeras longer than 110 nt (nucleotides). Finally, we got the positive dataset with 42,085 positive interactions with the labels of “1” (S1 Table).

There are more than 557 thousands of miRNA:target-gene interactions deposited in MirTarBase (Release 7.0) with strong or weak evidence. To generate the negative dataset, we made pseudo combinations of miRNA and gene avoiding the miRNA: gene pairs in MirTarBase. The cDNA sequences of genes in the pseudo combinations were retrieved from human genome (GRCh38) and mouse genome (GRCm38) using SAMtools [37]. The pseudo target sites were taken randomly from 3’UTR (untranslated region), 5’UTR or ORF (open reading frame) of genes with a proportion of 7:2:1 and the overall length of each pseudo miRNA:target chimera was set to be 110 nt (nucleotides). Altogether, we generated the negative dataset containing 94,764 human (S2 Table) and 22,531 mouse (S3 Table) miRNA:pseudo-target site interactions with the labels of “0”.

The negative and positive datasets were merged together and separated randomly into train (149,439), validation (4,941) and test (5,000) datasets. In the 10-fold cross validation (CV) experiments, the merged dataset was divided into 10 segments with about the same number of miRNA:target chimeras (15 938). In each experiment, nine segments were used for training while the remaining one was used for evaluating the performance of the model.

#### MiRNA:Target-gene datasets

To predict the target genes of miRNAs, the positive and negative experiment-validated miRNA:target-gene pairs were downloaded from MirTarBase and Diana TarBase respectively[35,38]. Next, we strictly selected the most convinced data. The positive dataset was composed of all the interactions of miRNAs and genes with strong evidence while the negative dataset only contained those with direct evidence of no interaction. The data appearing both in the positive and negative dataset were removed. The mature miRNA sequences were downloaded from miRBase database [39] and the transcripts sequences of genes were retrieved from human cDNA (complementary DNA) annotation file (Homo_sapiens.GRCh38.cdna.all.fa). Since there are many transcripts for one gene, we only use the longest transcript and the shortest transcripts to represent positive and negative interaction genes, respectively. All the interactions of miRNAs and genes which we failed to retrieve the sequences were removed from the datasets. The final experiment-validated positive dataset (S4 Table) contains 7815 items and negative dataset (S5 Table) contains 281 items.

### 2.2 Sequence padding and vectorization

Since the contextual sequence around the target site in the mRNA has great impact on the interaction [40], we considered both the direct interaction sequences and the contextual sequences in mRNAs for our mode. So, the miRNA:target-site chimeras contain the contextual sequences around the target sites, which keeps more information for the deep learning. Upon inspecting the positive miRNA:target-site chimeras, we selected the positive data with the length of no more than 110. Since different positive miRNA: target-site chimeras had different lengths, we padded each positive sequence into the length of 110 for batch learning in the next model training process. The padded sequences were randomly generated avoiding 4 continuous pairing bases with corresponding miRNAs. Next, we generated pseudo miRNA: target-site chimeras with each length of 110 nt to be the negative samples.

**Fig 1:**
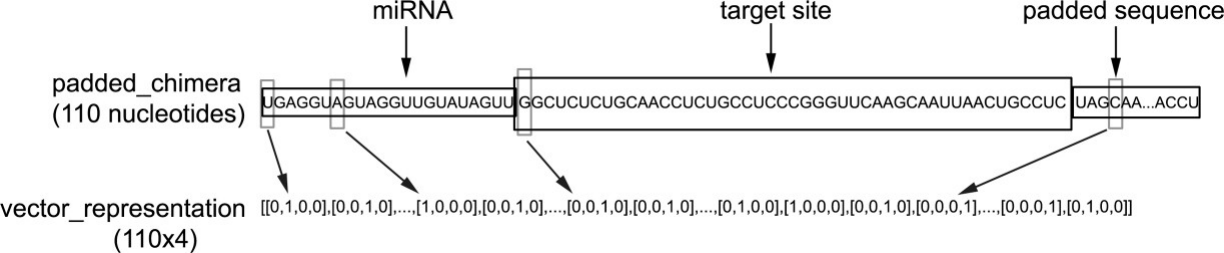
Schematic illustration of encoding the miRNA:target-site chimera. The miRNA and target sequence are concatenated and padded to 110 nucleotides/bases long. Using “one-hot” encoding, the padded chimera is represented by a 110 x 4 tensor.

To encode RNA sequences, the “one-hot” encoding was used for each base ("A":[1,0,0,0],"U":[0,1,0,0],"G":[0,0,1,0],"C":[0,0,0,1]). After encoding, each miRNA:target-site chimera can be represented by a 110 x 4 tensor, which was used in our supervised deep learning (Fig 1).

### 2.3 Architecture of the proposed multi-layer convolutional neural network

The designed architecture and parameters of the deep convolutional neural network (CNN) were showed in Table 1. In the model, the input sequences were first convolved by sixteen kernels with the size of 2 over a single spatial dimension (filters: 16, kernel size: 2) followed by max pooling. Second, the output tensors flowed through the second convolution layer (filters: 32, kernel size: 3) and max-pooling layer. And then there were the third convolution layer (filters: 64, kernel size: 4) and max-pooling layer, followed by the last convolution (filters: 128, kernel size: 5) and max-pooling layer. All the max-pooling layers took the maximum value with the size of 2.

**Table 1:** The structure and parameters of our CNN model.

Layer (type)	Output Shape	#Parameters
conv1d_1 (Conv1D)	(None, 110, 16)	144
max_pooling1d_1 (MaxPooling1D)	(None, 55, 16)	0
conv1d_2 (Conv1D)	(None, 55, 32)	1568
max_pooling1d_2 (MaxPooling1D)	(None, 28, 32)	0
conv1d_3 (Conv1D)	(None, 28, 64)	8256
max_pooling1d_3 (MaxPooling1D)	(None, 14, 64)	0
conv1d_4 (Conv1D)	(None, 14, 128)	41088
max_pooling1d_4 (MaxPooling1D)	(None, 7, 128)	0
flatten_1 (Flatten)	(None, 896)	0
dropout_1 (Dropout)	(None, 896)	0
dense_1 (Dense)	(None, 128)	114816
dropout_2 (Dropout)	(None, 128)	0
dense_2 (Dense)	(None, 1)	129

Total params: 166,001

After multi-layer convolution and max-pooling operations, all the extracted features were passed to a fully-connected layer (units: 128). The last layer is only one unit obtained by the Sigmoid activation function on the probability of the miRNA:target-site chimeras. The activation functions for other layers, if needed, are chosen to be “relu”. For generalization of our model, we added two dropout layers [41] before fully-connected layers as showed in Table 1 as well as L2 regularization [42] on the parameters of the fully-connected layer with 128 units. The total number of parameters was 166,001, mostly due to the fully-connected layers.

### 2.4 Model training and evaluation

The loss function we employed was the cross entropy [43] between the predicted values and the actual labels (“1” or “0”). The Adam optimizer was applied to learn the network weights in a back-propagation fashion [44]. During the training process, the generalization error was also monitored using validation dataset. The training process was not stopped until the loss on evaluation dataset did not decrease any more. After training, the learned parameters as well as the model structure were stored.

Using the trained model, we calculated the classifier performance on the test dataset in terms of sensitivity, specificity, F1-Score, Matthews Correlation Coefficient and accuracy. (TP: true positive, TN: true negative, FP: false positive, FN: false negative)

Sensitivity:

$$Sen.=\frac{TP}{TP+FN}$$ (1)

Specificity:

$$Spe.=\frac{TN}{TN+FP}$$ (2)

F1-Score:

$$F1=\frac{2*TP}{2*TP+FP+FN}$$ (3)

Matthews Correlation Coefficient (MCC):

$$MCC=\frac{TP*TN-FP*FN}{\sqrt{\left(TP+FN)*(TN+FP)*(TN+FN)*(TP+FP\right)}}$$ (4)

Accuracy:

$$Acc.=\frac{TP+TN}{TP+TN+FP+FN}$$ (5)

Also, we plotted the receiver-operating characteristic curve (ROC) with the calculated area under the ROC curve (AUC). With decreasing thresholds on the decision function used, corresponding false positive rates (fpr) and true positive rates (tpr) were computed. ROC curve was drawn based on a series of fpr and tpr.

### 2.5 Code implementation and availability

Because only a single candidate site to be classified as positive is required for long target sequences, the model is particularly sensitive to false positive. Hence, we filtered out the candidate sites of mRNA using RNAhybrid with the minimum free energy (MFE) of miRNA:target duplexes < = -20 kcal/mol [45].

The implemented cnnMirTarget was based on the well-trained CNN model using the training dataset and can be used to predict whether miRNAs can target candidate sequences or even full length of mRNAs. The cnnMirTarget takes the inputs of miRNAs and the target sequences as parameters. If the target sequence provided is less than 110, it will be padded into 110 with the same strategy described in the methods 2.2. For long mRNA sequences, all the candidate sites are filtered out using RNAhybrid followed by prediction using the trained CNN model. The final prediction result is based on the maximum prediction value of all the candidate sites. If the output value is greater than the threshold of 0.5, the prediction result is “True”, and otherwise “False”.

The cnnMirTarget source code, which is written in python with keras library, is freely available through GitHub (https://github.com/zhengxueming/cnnMirTarget).

### 2.6 Prediction performance comparison with other methods

We next tested the performance of the cnnMirTarget and compared with that of other state-of-the-art target prediction software tools on the experiment-validated positive and negative miRNA:target-gene datasets (see section 2.1). All the predicted datasets of miRTarget3 (miRDB v5.0), metaMIR and TargetScan (v7.0) were downloaded from each website and searched for each miRNA:target-gene pair in the positive and negative interactions [46–49]. Since the miRTarget3 and TargetScan had no negative datasets available, the miRNA:target-gene pairs not found in their interaction datasets were considered as negative. Based on the searched results, the performance was evaluated by sensitivity, specificity, F1-Score, MCC and accuracy.

## 3 Results

### 3.1 Performance of our model on the test miRNA:Target-site dataset

For the training/evaluation/test dataset splitting, the model was trained on the training dataset with enough epochs, evaluated on the evaluation dataset and finally the performance was tested on the test dataset. In the 10-fold CV, we trained our model with the nine folds while the remaining one fold was used for testing the performance in each time. For conciseness, we showed the average performance along with standard error (SE) for the 10-fold CV experiments (Table 2).

**Table 2:** Performance of our CNN model to predict miRNA:Target-site interactions.

Dataset partition methods	Sen.(%)	Spe.(%)	F1(%)	MCC(%)	Acc.(%)
train/evaluation/test	94.82	98.26	94.97	93.18	97.36
10-fold CV	93.95± 2.91	97.16± 1.25	94.76± 0.86	92.2± 1.02	97.11± 0.52

The performance on the test dataset (row two) and one-fold dataset (row three) unseen in the training processes was shown as sensitivity (column 2), specificity (column 3), F1-Score (column 4), MCC (column 5) and accuracy (column 6). For the 10-fold CV, the performance was shown as mean ± standard error (SE).

As shown, we got similar values of sensitivity (column 2), specificity (column 3), F1-score (column 4), MCC (column 5) and accuracy (column 6) for different dataset splitting strategies in our model. In the training/evaluation/test dataset splitting, the overall accuracy of prediction is 97.36% and all the other values are more than 93%, indicating high generalization performance of our trained model.

**Fig 2:**
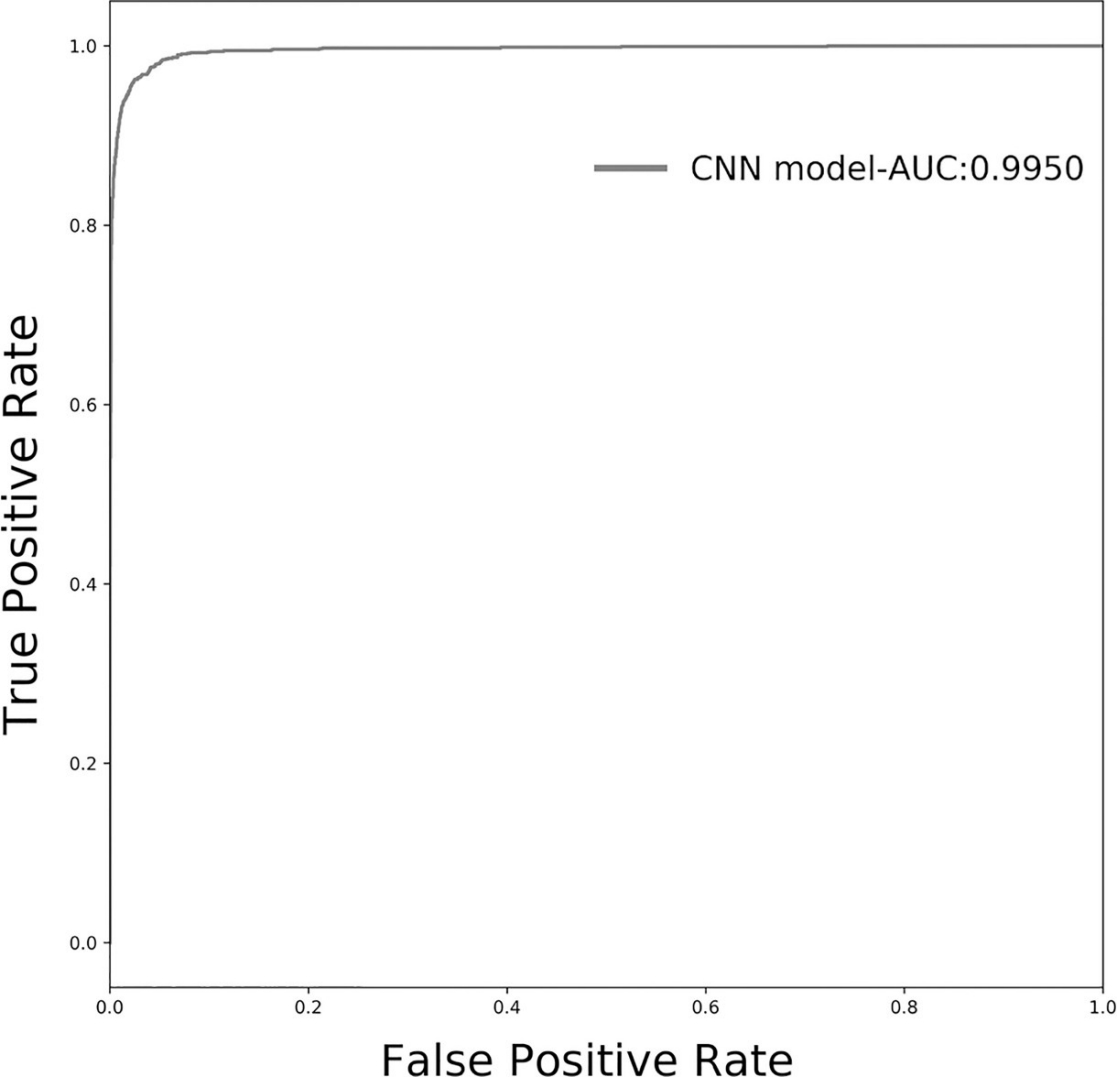
The ROC curve of our CNN model. The model trained on the training dataset was used to predict the test dataset. The ROC curve was plotted based on the prediction results of the test dataset. The area under the ROC curve (AUC) was calculated and showed.

To further evaluate our model, we plotted the ROC curve of prediction on the test dataset. As shown in Fig 2, the AUC of ROC curve is 99.50%, indicating high performance for recognizing the target sequences of the miRNAs.

### 3.2 Comparison with other methods

Since our CNN model showed high performance to predict the target sites of miRNAs, we wanted to test the performance and compare with other methods on predicting target genes of miRNAs. Due to the long length of mRNAs, there was great possibility of false positive as described in the methods 2.5. So, we introduced a filter step to find the candidate sites in the cnnMirTarget. To get the most reliable interaction dataset of miRNAs and genes, we carefully selected the experiment-validated positive and negative datasets as described in methods 2.1. Because of the small number of negative data available, the merged dataset is highly imbalanced. To classify targets or non-targets of miRNAs, the threshold we used here is 0.5.

The prediction performance for miRTarget3, metaMIR, TargetScan7 as well as our cnnMirTarget was evaluated on the experiment-validated positive and negative miRNA:target-gene datasets (described in section 2.1). The prediction results for miRTarget3, metaMIR, TargetScan7 as well as our cnnMirTarget were showed in the Table 3. The true positive and true negative interactions predicted by our cnnMirTarget are 5,179 and 162 respectively. Compared with miRTarget3 and TargetScan7, cnnMirTarget has better performance on the positive dataset (Column 2). On the other hand, cnnMirTarget has better performance on the negative dataset than metaMIR (Column 3).

**Table 3:** Prediction performance of different algorithms on the experiment-validated miRNA: Gene interactions.

Algorithm	#predicted positive	#predicted negative	Sen.(%)	Spe.(%)	F1(%)	MCC(%)	Acc.(%)
cnnMirTarget	5179	162	66.27	57.65	78.99	9.21	65.97
miRTarget3	1000	263	12.80	93.59	22.64	3.53	15.60
metaMIR	5131	72	65.66	25.62	78.01	-3.37	64.27
TargetScan7	1455	272	18.62	96.80	31.36	7.33	21.33

The experiment-validated positive dataset contains 7815 interactions, while the negative dataset contains 281 pseudo-interactions. Column 2: number of predicted positive interactions in the positive dataset for each algorithm. Column 3: number of predicted negative interactions in the negative dataset for each algorithm. Column 4–8: Sensitivity (Sen.), Specificity (Spe.), F1-Score, MCC, Accuracy (Acc.).

Although miRTarget3 and TargetScan7 have high specificity, they missed many true miRNA:gene interactions (low sensitivity: 12.80% and 18.62%, respectively). And metaMIR has comparably higher sensitivity, but with many false positives (low specificity: 25.62%). Overall, our algorithm outperforms other methods for predicting miRNA-mRNA interactions indicated by F1-Score, MCC as well as accuracy.

## 4. Discussion

Different from traditional machine learning algorithms, deep learning can automatically extract patterns from canonical and non-canonical pairing between the miRNAs and its targets. In this study, we used four layers of convolution followed by max-pooling operations in our model, which extract features hierarchically from miRNA:target-site chimeras. By using two dropout layers and the L2 regularization in the first dense layer, our model showed little generalization error on the evaluation dataset during the training process. The high performance of trained model on test dataset showed the learned features can be used to predict the interaction sites of miRNAs with high accuracy. In summary, our trained CNN-based model can predict the interaction of miRNAs:target-sites with high performance.

In machine learning especially deep learning, it is vital important to collect large amounts of reliable data. For miRNA targets prediction, different methods used different datasets to train their models. So, there is little overlap among them, which makes the prediction of miRNA targets difficult and challenging. In this study, we only chose the experiment-validated data mainly from high-throughput sequencing. Different from other rule-based machine learning methods, the convolutional neural network need equal amount of positive and negative datasets to train the model. Since it is harder to collect the data of negative miRNA:target-site interactions, we generated a large negative dataset as described in methods.

Although our methods showed great performance on the test dataset to predict target sites, the accuracy to predict target genes of miRNAs was dramatically decreased. Since one gene can express different transcripts under different conditions, an experiment-validated miRNA:target-gene interaction does not mean that the miRNA can target any transcript of the gene. In fact, we found much controversial data appearing both in positive and negative experiment-validated miRNA:target-gene interaction dataset.

Different from predicting the target sites of miRNAs, there are harder to predict the interactions of miRNAs and genes because of many other factors involved the process. For an example, the secondary structure of mRNA may affect the accessibility of miRNA [50]. Moreover, the stability of miRNA:target hybrids has great importance on the interactions [45]. Although we filtered out the candidate sites using RNAhybrid, the minimum free energy (MFE) should be carefully selected. Also, there exist many weak binding sites in some mRNA. So, the synergistic effects should be considered [51].

Furthermore, there are complicated molecular interaction networks in the cell, which affect the interactions of miRNAs and target mRNAs. The binding sites on mRNA can be occupied by other proteins or RNAs and the miRNA:mRNA interactions may be eliminated by circRNAs [52]. Also, Ago protein binding sites in mRNA may bring mRNAs to miRNAs, which leads to interactions [53]. In brief, there are many factors that can affect the interaction of miRNAs and mRNAs, which should be taken into consideration to improve our model in the future.

So far, there are tens of computational prediction algorithms to predict miRNAs targets [54]. But, owing to the great difference of prediction results for different predictors, the prediction of miRNA target is still a challenge. Here, we designed the CNN model to learn both canonical and non-canonical interactions automatically from experiment validated miRNA:target-sites chimeras. The results showed that our model is very successful to predict the target sites of miRNA, but there are great room to improve the performance on predicting miRNA:target-gene interaction.

## Supporting information

S1 Table Positive dataset of miRNA:Target-site. (XLSX)

S2 Table Negative dataset of miRNA:Target-site from human. (XLSX)

S3 Table Negative dataset of miRNA:Target-site from mouse. (XLSX)

S4 Table Positive dataset of miRNA:Target-gene. (XLSX)

S5 Table Negative dataset of miRNA:Target-gene. (XLSX)
